# Body composition in prepubertal children with idiopathic premature adrenarche: implications for cardiometabolic health

**DOI:** 10.1038/s41390-024-03776-2

**Published:** 2024-12-18

**Authors:** Asaf Ben Simon, Michal Yackobovitch-Gavan, Adi Uretzky, Anat Segev-Becker, Liat Perl, Eyas Midlij, Ophir Borger, Avivit Brener, Yael Lebenthal

**Affiliations:** 1 The Institute of Pediatric Endocrinology, Diabetes and Metabolism, Dana-Dwek Children’s Hospital, Tel Aviv Sourasky Medical Center, Tel Aviv, Israel affiliated to the Faculty of Medicine, Tel Aviv University, Tel Aviv, Israel; 2 Department of Epidemiology and Preventive Medicine, School of Public Health, Faculty of Medicine, Tel Aviv University, Tel Aviv, Israel; 3 The Nutrition and Dietetics Unit, Tel Aviv Sourasky Medical Center, Tel Aviv, Israel

## Abstract

**Background:**

Premature adrenarche (PA) has been reported as a potential precursor of metabolic disease. We aimed to explore the interaction between body composition and cardiometabolic health of prepubertal children with PA.

**Methods:**

This observational study comprised of 87 children with PA (15 boys, 8.0 ± 1.2 years) and 87 healthy sex- and age-matched controls. Body composition was measured by bioelectrical impedance analysis.

**Results:**

Children with PA had a higher median BMI z-scores (*P* = 0.001), higher median fat percentage (*P* = 0.006*)*, and lower muscle-to-fat z-scores (*P* = 0.050) compared to controls. There were no significant group differences in blood pressure percentiles and lipid profiles. Fat percentage of children with PA was positively correlated and the MFR z-score was negatively correlated with: BMI z-score, systolic blood pressure percentile, and atherogenic dyslipidemia index (*P* < 0.001 for all). There were significant correlations between parental and offspring MFR z-scores in the control group (father-child: r = 0.528, *P* = 0.020; mother-child: r = 0.359, *P* = 0.031), but none in the PA group.

**Conclusions:**

Children with PA exhibited an unfavorable body composition in association with their metabolic health even before the onset of puberty. Furthermore, while healthy children displayed heritable body composition traits, children with PA did not, possibly suggesting a relatively greater role for environmental factors in the PA group.

**Impact:**

- Prepubertal children with premature adrenarche have a low muscle-to-fat ratio compared to healthy sex- and age- matched controls.
- The body composition of prepubertal children with premature adrenarche is associated with their systolic blood pressure percentile and atherogenic dyslipidemia index.
- Children with premature adrenarche and healthy weight exhibited higher adiposity compared to healthy weight controls, and those with overweight/obesity showed higher rates of elevated blood pressure and higher dyslipidemic atherogenic indices compared to overweight/obesity controls.
- These findings highlight the importance of early identification, intervention, and lifestyle changes for children with premature adrenarche to help prevent cardiometabolic complications.

## Introduction

Adrenarche is characterized by a physiological rise in adrenal androgen production during early childhood. It is driven by changes in adrenal response to the adrenocorticotropic hormone (ACTH), resulting in increased secretion of dehydroepiandrosterone sulfate (DHEAS).^1^ Clinical manifestations of adrenarche include the onset of pubic hair (pubarche) and the development of sebaceous glands (resulting in oily skin and hair) and apocrine glands (causing body odor). Premature pubarche refers to the clinical appearance of sexual hair before 8 years of age in girls and before 9 years of age in boys.^2^ Premature adrenarche (PA) refers to premature pubarche and/or other related clinical signs of adrenarche accompanied by elevated DHEAS levels. This diagnosis is made when there is no evidence of gonadarche or overt virilization, and after excluding androgen-producing tumors, steroidogenic enzyme defects, and central precocious puberty.^1,3^ Historically considered a benign normal variant of puberty, PA has since been reported as a potential precursor to polycystic ovarian syndrome (PCOS), and insulin resistance in later life, sometimes already recognizable in childhood or adolescence.^1,2,4,5^ Given those associations, it is reasonable to consider that PA may also be a forerunner of metabolic syndrome (MetS).

MetS comprises a cluster of cardiovascular risk factors, central adiposity, abnormalities in glucose and lipid metabolism, and elevated blood pressure (BP).^6^ Cardiovascular diseases (CVD) are a major public health concern, with greater morbidity and mortality with increasing age among adults.^7^ A direct connection between childhood‐onset cardiovascular risk factors and CVD during young adulthood has been reported,^8^ including studies that focused upon obesity as a cause.^9^

The routine standard of care of our Pediatric Endocrine Institute includes identifying and mitigating modifiable CVD risk factors. Since 2018, all patients referred for consultation undergo a comprehensive metabolic risk assessment, including body composition measurement by bioimpedance analysis (BIA).^10^ Our group recently reported the predictive value of muscle-to-fat ratio (MFR) z-scores in assessing CVD risk factors, where an imbalance between muscle and fat tissue increases the risk for early-onset cardiometabolic derangements in youth with overweight and obesity,^11^ with type 1 diabetes^12^ and with non-classic congenital adrenal hyperplasia,^13^ while an increase in adiposity predicted cardiometabolic risk in young patients with celiac disease.^14^

It has been speculated that premature adrenarche could pose an increased risk of obesity and metabolic derangements in adolescence and early adulthood.^5^ However, little is known about the body composition of children with premature adrenarche and its association with unfavorable metabolic outcomes or with the implications for cardiometabolic health. ^15–17^We aimed to describe the body composition of prepubertal children with premature adrenarche and its interaction with metabolic syndrome components.

## Methods

### Study design and population

This real-life, observational study included prepubertal pediatric subjects aged 5 to 10 years who underwent BIA assessment at our endocrine institute in a tertiary medical center between January 2020 and March 2023. We queried the BIA database to identify patients diagnosed with PA (study group) and those being monitored for growth concerns (control group). Electronic medical records for both groups were thoroughly reviewed. All children in the PA group met the following inclusion criteria: onset of pubic and/or axillary hair before age 8 in girls and age 9 in boys, absence of gonadarche (Tanner stage 1 breast/testicular development), elevated DHEAS levels, and no evidence of adrenal enzymatic defects or androgen-producing tumors. Referrals for the children in the study group came from pediatricians due to concerns about premature pubic and/or axillary hair, acne, or body odor. Children in the control group were referred due to parental concerns about stature. Following evaluation by a senior pediatric endocrinologist, who excluded any endocrine or other medical conditions, these children were classified as healthy prepubertal controls. The exclusion criteria were chronic medical conditions, medications affecting body composition, severe underweight (body mass index [BMI] z-score ≤ −2.25 standard deviations [SD]), short stature (height z-score ≤ −2 SD), metabolic bone disease, malignancies, genetic syndromes, bone dysplasia, non-classic congenital adrenal hyperplasia, and other endocrine disorders. The study group consisted of 87 children (15 boys) diagnosed with premature adrenarche, while the control group comprised 87 healthy, sex- and age-matched children.

### Clinical evaluation

The comprehensive clinical evaluation for patients referred to the endocrine institute includes a detailed anamnesis and clinical assessment. Each child undergoes anthropometric measurements, which consist of height (measured with a Harpenden stadiometer manufactured by Holtain Ltd., Crosswell, UK) and weight assessed by bioelectrical impedance analysis while dressed in light clothing. A registered pediatric nurse measures blood pressure (BP) using the Welch Allyn Vital Signs Monitor VSM 300 (Welch Allyn, Inc., Beaverton, OR) and the appropriate cuff. The accompanying parent’s height is also measured, a BIA assessment is offered, and the reported height of the absent parent is recorded.

### Measurement of Body Composition

The Tanita Body-Composition Analyzer (Tanita MC-780 MA and GMON Professional Software) was used to indirectly measure body composition. The BIA measures fat and muscle of the whole body as well as body segments (trunk, upper and lower limbs). This method has been clinically validated as being accurate and reliable, and to provide highly reproducible results.^18,19^ For the bioelectrical impedance analysis (BIA) measurement, subjects stood barefoot on the analyzer and held onto the device’s handles. The entire process was completed in about one minute for each individual.

### Biochemical analysis

Children with clinical evidence of premature adrenarche underwent a hormonal evaluation that included a serum basal androgen profile (dehydroepiandrosterone sulfate [DHEAS], androstenedione [Δ4A], testosterone, and 11-deoxicortisol [compound-S] when indicated), serum basal and post-corticotropin (0.25 mg, IV Synacthen, Ciba-Geigy, Basel, Switzerland) stimulated 17 hydroxyprogesterone (17OHP), and cortisol in order to exclude an adrenal enzymatic defect. Measurements of serum fasting glucose concentrations, insulin concentrations and lipid profile tests were performed as part of the routine standard of care. Serum DHEAS and androstenedione levels were measured using an automated chemiluminescence assay (Immulite 2000, Siemens Healthcare Diagnostics Product Ltd., UK). Testosterone levels were assessed by electrochemiluminescence (Roche, Cobas E 601). 17OHP was measured with a direct enzyme immunoassay (DBC, Diagnostic Biochem Canada Inc). Cortisol levels were quantified using the Coat-A-Count radioimmunoassay (Diagnostics Products Corporation, Los Angeles, CA). Glucose was measured using the glucose oxidase colorimetric method (Hitachi 917 automated analyzer, Roche Diagnostics, Mannheim, Germany), insulin was assessed with the electrochemiluminescence immunoassay (Roche Diagnostics GmbH, Mannheim, Germany), and total cholesterol (TC), triglycerides (TG), and high-density lipoprotein (HDL) concentrations were measured with the enzymatic colorimetric method (Hitachi 904 automated analyzer, Roche Diagnostics). Our medical center holds JCI accreditation, ensuring that our laboratories meet the highest standards of quality in healthcare services.

### Data collection

The patients’ medical files are stored electronically, with laboratory data accessible from the individual’s health maintenance organization. Sociodemographic information, perinatal history, medical conditions, medications, and family history of cardiovascular risk factors were obtained from the medical records. At the time of BIA assessment, clinical data were collected, including anthropometric measurements, vital signs, pubertal status, and laboratory results.

### Definition of study variables

Socioeconomic position (SEP) by home address was analyzed based on the Israel Central Bureau of Statistics’ Characterization and Classification of Statistical Areas within Municipalities and Local Councils by the Socio-Economic Level of the Population.^20^ Birth weight z-scores adjusted for gestational age were calculated by PediTools Electronic Growth Chart Calculators based on the Fenton growth chart for preterm infants.^21^ Corrected birth weight z-scores were used to define appropriate for gestational age (AGA) as scores from −1.645 to 1.645, small for gestational age (SGA) as scores below −1.645, and large for gestational age (LGA) as scores above 1.645.^22^

BMI was computed by dividing weight in kilograms by height in meters squared. CDC 2000 growth charts were used to convert the patients’ height, weight, and BMI values into sex- and age-specific standard deviation scores (z-scores).^23^ Overweight was defined as a BMI percentile ≥85^th^ and <95^th^ (1.04 ≤ z-score < 1.65), and obesity as a BMI percentile ≥95^th^ percentile (z-score ≥1.65).^24^ The mid-parental height (MPHt) was calculated as follows: (paternal height [cm] + maternal height [cm] ± 13 cm)/2, and the derived MPHt z-scores were calculated.^25^ The delta height z-score represents the linear growth compared to the potential genetic height and it was calculated as the difference between the subjects’ height z-scores and their MPHt z-scores. Systolic and diastolic BP percentiles were determined using an online pediatric blood pressure calculator based on sex, age, and height.^26^

Appendicular skeletal muscle mass (ASMM) was calculated as the total muscle mass of the four limbs, while the MFR was determined using the formula MFR = ASMM [kg]/fat mass [kg]. BIA pediatric reference curves were used to calculate the z-scores for the MFR.^19^ The trunk-to-appendicular fat ratio was calculated as trunk fat mass divided by appendicular fat mass (=the sum of the fat mass of 4 limbs).^27^

Fasting plasma TG concentrations of ≥110 mg/dL were considered elevated, and an HDL ≤ 40 mg/dL was considered low.^6^ Lipid profile z-scores were calculated according to sex and weight status using Israeli population references.^28^ Homeostatic Model Assessment of Insulin Resistance (HOMA-IR) was calculated as follows: insulin (mU/L) x glucose (mg/dL)/ 405.^29^ Glycated hemoglobin (HbA1c) and HOMA-IR levels were compared to sex- and age-matched values in European children and taken as being elevated when they were at or above the 90^th^ percentile.^30^

### Ethics

The study was approved by the Ethics Committee of the Tel-Aviv Sourasky Medical Center (TLV-0846-20) according to the Helsinki Declaration. Informed parental consent was waived. The data were processed in alignment with Good Clinical Practice standards.

### Statistical analyses

Statistical analysis was carried out using Statistical Package for the Social Sciences software, version 28 (SPSS Inc., Chicago, IL). All tests were two-tailed. Normality of the continuous variables was evaluated using the Shapiro-Wilk test. Normally distributed data are reported as mean ± standard deviation (SD), while non-normally distributed data are expressed as median and interquartile range [IQR]. Pearson’s chi-square test compared categorical variables between the PA and control groups. For continuous data, independent sample t-tests or Mann-Whitney U tests were applied, depending on normality. The Spearman correlation (due to skewed distribution of one or both of the correlated variables) was applied to assess the relationship between body composition parameters (body fat percentage [FATP], truncal-to-appendicular fat ratio, and MFR z-score) and MetS components (BMI z-score, BP percentiles, glucose concentrations, TG z-scores, HDL z-scores, TG/HDL ratio) as well as parent-offspring MFRs. Statistical significance was defined as *P* ≤ 0.05.

## Results

The entire cohort comprised 174 prepubertal children (87 PA and 87 healthy controls) with a mean age at BIA measurement of 8.1 ± 1.2 years. Their collective SEP was similar and above average (median SEP cluster of 8, range 3–10). The PA group was significantly more likely to have a family history of cardiometabolic disease (obesity, hypertension, type 2 diabetes, dyslipidemia, cardiovascular disease) compared to the healthy controls (68.2% vs 45.9%, respectively, *P* = 0.003).

The pregnancy and perinatal characteristics of both groups are presented in Supplementary Table 1. The overall perinatal characteristics were similar for both groups, 26.4% of the PA children were conceived following assisted reproduction, 9.6% of their mothers had PCOS, and 8.2% PA children had in utero exposure to gestational diabetes mellitus. Around 80% of the entire cohort were born at term with an AGA birthweight, while in the PA group 15.7% were born SGA compared to 11.9% of the controls (*P* = 0.701).

The anthropometric characteristics and body composition parameters of the study group and their controls are presented in Table 1. The children in the PA group had a higher median height z-score compared to the controls (0.59 [IQR −0.08, 1.35] vs. −0.13 [IQR −0.79, 0.77], *P* < 0.001, respectively), greater mean delta height z-score (the subjects’ height z-scores minus their MPHt z-scores, 0.56 ± 0.94 vs. −0.14 ± 0.94, *P* < 0.001), and higher BMI z-scores (0.52 [IQR −0.22, 1.44] vs. −0.07 [IQR −0.93, 0.83], *P* = 0.001).

**Table 1:** Anthropometric characteristics and body composition parameters of the premature adrenarche group and their healthy controls.

	Premature adrenarche *n* = 87	Healthy controls *n* = 87	*P* value
Girls (%)	72 (82.8)	72 (82.8)	
Age, years	8.0 ± 1.2	8.2 ± 1.3	0.364
Anthropometrics			
Height, *z-score*	0.59 [−0.08, 1.35]	−0.13 [−0.79, 0.77]	**<0.001**
Mid-parental height (MPHt), *z-score*	0.04 ± 0.79	0.17 ± 0.78	0.295
Delta height, *z-score*	0.56 ± 0.94	−0.14 ± 0.94	**<0.001**
Body mass index, *z-score*	0.52 [−0.22, 1.44]	−0.07 [−0.93, 0.83]	**0.001**
Body composition parameters			
Fat, *percentage*	24.9 [21.7, 27.3]	21.9 [20.2, 26.5]	**0.006**
Truncal fat mass, *%*	18.4 [15.8, 21.4]	16.4 [14.3, 21.0]	**0.027**
Truncal fat mass, *kg*	3.1 [2.4, 4.1]	2.5 [2.0, 3.7]	**0.006**
Appendicular fat mass, *kg*	3.8 [3.3, 5.4]	3.4 [2.7, 4.5]	**0.003**
Trunk-to-appendicular fat ratio	0.80 [0.71, 0.85]	0.82 [0.70, 0.88]	0.258
Muscle-to-fat ratio (MFR), *z-score*	−0.82 [−1.19, −0.28]	−0.38 [−1.08, 0.06]	**0.050**
Physical activity, hrs/wk	2 [1,4]	2.75 [1,4]	0.798
Family history, *n* (%)	*n* = 85	*n* = 85	
Obesity	22 (25.9)	12 (14.1)	0.055
Hypertension	13 (15.3)	18 (21.2)	0.321
Type 2 diabetes	32 (37.6)	34 (40.0)	0.753
Dyslipidemia	13 (15.3)	15 (17.6)	0.679
Cardiovascular disease	30 (35.3)	19 (22.4)	0.063
Any cardiometabolic disease	58 (68.2)	39 (45.9)	**0.003**

Data are expressed as number (percent), mean ± standard deviation or median [interquartile range]. The delta height z-score represents the linear growth compared to the potential genetic height and it was calculated as the difference between the subjects’ height z-scores and their MPHt z-scores.

Bold values denote statistical significance at the *P* ≤ 0.05 level.

The body composition of the children in the PA group was characterized by higher total fat percentage compared to the healthy controls (24.9 [IQR 21.7, 27.3] vs. 21.9 [IQR 20.2, 26.5], respectively, *P* = 0.006), higher truncal fat percentage (18.4 [IQR 15.8, 21.4] vs. 16.4 [IQR 14.3, 21.0], *P* = 0.027), higher truncal fat mass (3.1 [IQR 2.4, 4.1] vs. 2.5 [IQR 2.0, 3.7], *P* = 0.006), higher appendicular fat mass (3.8 [IQR 3.3, 5.4] vs.3.4 [IQR 2.7, 4.5], *P* = 0.003), and lower MFR z-scores (−0.82 [IQR −1.19, −0.28] vs. −0.38 [IQR −1.08, 0.06], *P* = 0.050). There were no group differences in the trunk-to-appendicular fat ratio or parent-reported physical activity levels, and both the study and control groups had negative correlations between their MFR z-scores and their trunk-to-appendicular fat ratios (r = −0.669, *P* < 0.001 and r = −0.468, *P* < 0.001, respectively).

The median basal androgen levels of prepubertal children with PA were as follows: DHEAS 1.55 [IQR 0.87, 2.51] µmol/L, androstenedione 0.52 [IQR 0.50, 1.50] nmol/L, testosterone 0.21 [IQR 0.20, 0.33] nmol/L, 17OHP 1.40 [IQR 0.90, 2.30] nmol/L and cortisol 244 [IQR 178, 317] nmol/L.

The MetS components of the study and control groups are listed in Table 2. There were no significant group differences in the rates of obesity, elevated BP, or elevated fasting glucose and dyslipidemia (elevated TG and low HDL concentrations). The mean HbA1c level of the PA group was 5.4 ± 0.3% (35.5 mmol/mol) and the mean HOMA-IR level was 2.4 ± 1.3; 66% of the PA children had elevated HbA1c and/or HOMA-IR levels.

**Table 2:** Metabolic syndrome components of the premature adrenarche group and their healthy controls.

	Premature adrenarche *n* = 87	Healthy controls *n* = 87	*P* value
Overweight/obese	28 (32.2)	19 (21.8)	0.123
Overweight: BMI ≥85^th^ and <95^th^ centile	11 (12.6)	10 (11.5)	0.824
Obese: BMI ≥95^th^ centile	17 (19.5)	9 (10.3)	0.089
Blood pressure			
Systolic, *mmHg*	106 [99, 113]	104 [99, 109]	0.202
Systolic, *%*	78 [50,92]	74 [58,90]	0.796
Diastolic, *mmHg*	63 [59,70]	62 [57,67]	0.136
Diastolic, *%*	68 [54,84]	63 [46,80]	0.178
Blood pressure categories, *n (%)*			
Elevated systolic, ≥95^th^ centile	11 (12.6)	11 (12.6)	
Elevated diastolic, ≥95^th^ centile	2 (2.3)	2 (2.3)	
Elevated systolic and/or diastolic, ≥95^th^ centile	13 (14.9)	11 (12.6)	0.661
Glucose, *mg/dL*	85 [80,89]	86 [82,90]	0.413
Lipid profile			
Dyslipidemia, elevated TG (TG > 110 mg/dL), *n (%)*	15 (17.2)	9 (10.3)	0.188
Dyslipidemia, low HDL (HDL < 40 mg/dL), *n (%)*	6 (6.9)	7 (8.0)	0.783
TG:HDL cholesterol ratio	1.29 [0.89, 1.89]	1.13 [0.92, 1.64]	0.748

Data are expressed as number and (percent) or median [interquartile range].

*TG* triglycerides, *HDL* high-density lipoproteins.

The correlations between HOMA-IR and the following parameters reached levels of significance: BMI z-score (r = 0.604, *P* < 0.001), FATP (r = 0.564, *P* = 0.002), MFR z-score (r = -0.456, *P* = 0.017), and DHEAS concentrations (r = 0.664, *P* = 0.001). The correlation between the DHEAS concentrations and FATP was also significant (r = 0.251, *P* = 0.036). Birthweight z-scores, however, were not significantly correlated with BMI z-scores, FATP, MFR z-scores, or HOMA-IR levels and DHEAS concentrations. Laboratory characteristics are shown in Supplementary Table 2.

### Subgroup analyses of children with pa stratified by weight status (Table 3)

Children with PA and overweight/obesity had higher height z-scores (*P* = 0.007), higher delta height z-scores (*P* = 0.022), higher fat percentages (*P* < 0.001), higher truncal fat mass (*P* < 0.001), higher truncal fat percentages (*P* < 0.001), higher appendicular fat mass (*P* < 0.001) and lower MFR z-scores (*P* < 0.001) compared to children with PA and healthy weight status. Comparative analysis of MetS components among children with PA and overweight/obesity revealed higher rates of elevated systolic BP (*P* < 0.001), dyslipidemia (low HDL, *P* = 0.006) and higher TG:HDL ratios (*P* < 0.001). No differences were found between PA subgroups in family history of cardiometabolic disease.

**Table 3:** Subgroup analyses within the premature adrenarche group according to weight status.

	Overweight/obese *n* = 28	Healthy weight *n* = 59	*P* value
Girls (%)	21 (75)	51 (86.4)	0.187
Age, years	8.2 ± 1.4	7.9 ± 1.1	0.311
Anthropometrics			
Height, *z-score*	1.05 [0.43, 1.50]	0.36 [−0.27, 1.18]	**0.007**
Mid-parental height (MPHt), *z-score*	0.11 ± 0.86	0.01 ± 0.77	0.625
Delta height, *z-score*	0.92 ± 0.97	0.40 ± 0.89	**0.022**
Body mass index, *z-score*	1.73 [1.54, 1.91]	0.17 [−0.45, 0.56]	**<0.001**
Body composition parameters			
Fat, *percentage*	31.3 [21.2, 35.1]	22.7 [20.8, 25.0]	**<0.001**
Truncal fat mass, *%*	25.6 [21.5, 29.3]	17.0 [14.3, 19.1]	**<0.001**
Truncal fat mass, *kg*	5.8 [4.0, 6.8]	2.7 [2.2, 3.2]	**<0.001**
Appendicular fat mass, *kg*	6.7 [5.1, 8.4]	3.4 [3.1, 3.8]	**<0.001**
Trunk-to-appendicular fat ratio	0.83 [0.74, 0.86]	0.78 [0.67, 0.83]	0.069
Muscle-to-fat ratio (MFR), *z-score*	−1.36 [−1.59, −1.18]	−0.47 [−0.88, −0.06]	**<0.001**
Blood pressure			
Systolic, *mmHg*	113 [109, 116]	103 [96, 109]	**<0.001**
Systolic, *%*	91 [83,94]	71 [43,85]	**<0.001**
Diastolic, *mmHg*	69 [65,71]	61 [57,67]	**0.001**
Diastolic, *%*	83 [64,86]	64 [48,81]	**0.013**
Blood pressure categories, *n (%)*			
Elevated systolic, ≥95^th^ centile	16 (57.1)	12 (20.3)	**<0.001**
Elevated diastolic, ≥95^th^ centile	6 (21.4)	5 (8.5)	0.089
Elevated systolic and/or diastolic, ≥95^th^ centile	16 (57.1)	14 (23.7)	**0.002**
Glucose, *mg/dL*	88 [82,90]	84 [80,88]	0.061
Lipid profile	n = 25	n = 45	
Dyslipidemia, elevated TG (TG > 110 mg/dL), *n (%)*	7 (28)	5 (10.6)	0.060
Dyslipidemia, low HDL (HDL < 40 mg/dL), *n (%)*	4.0 (16.0)	0 (0.0)	**0.006**
TG:HDL cholesterol ratio	1.72 [1.49, 2.37]	1.07 [0.75, 1.36]	**<0.001**

Data are expressed as number (percent), mean ± standard deviation or median [interquartile range]. The delta height z-score represents the linear growth compared to the potential genetic height and it was calculated as the difference between the subjects' height z-scores and their MPHt z-scores.

Bold values denote statistical significance at the *P* ≤ 0.05 level.

*TG* triglycerides, *HDL* high-density lipoproteins.

### Comparative analyses of children with healthy weight status (PA vs. control, Table 4)

The comparison between children with healthy weight in the PA group and those in the control group revealed that the PA group had higher height z-scores (*P* < 0.001), delta height z-scores (*P* < 0.001), higher fat percentages (*P* = 0.007), truncal fat mass (*P* = 0.009), truncal fat percentages (*P* = 0.031), and higher appendicular fat mass (*P* = 0.010). MFR z-scores tended to be lower for the healthy weight subgroup of children with PA compared to the control group, with no differences observed in the rates of MetS components.

**Table 4:** Subgroup analyses of children with healthy weight status: premature adrenarche vs. healthy controls.

	Healthy weight premature adrenarche *n* = 59	Healthy weight controls *n* = 68	*P* value
Girls (%)	51 (86.4)	56 (82.4)	0.528
Age, years	7.9 ± 1.1	8.3 ± 1.3	0.145
Anthropometrics			
Height, *z-score*	0.36 [−0.27, 1.18]	−0.38 [−0.93, 0.51]	**<0.001**
Mid-parental height (MPHt), *z-score*	0.01 ± 0.77	0.14 ± 0.76	0.371
Delta height, *z-score*	0.40 ± 0.89	−0.34 ± 0.89	**<0.001**
Body mass index, *z-score*	0.17 [-0.45, 0.56]	−0.29 [−1.24, 0.16]	**0.002**
Body composition parameters			
Fat, *percentage*	22.7 [20.8, 25.0]	21.2 [19.7, 23.7]	**0.007**
Truncal fat mass, *%*	17.0 [14.3, 19.1]	15.4 [13.6, 17.9]	**0.031**
Truncal fat mass, *kg*	2.7 [2.2, 3.2]	2.3 [1.88, 2.8]	**0.009**
Appendicular fat mass, *kg*	3.4 [3.1, 3.8]	3.1 [2.5, 3.7]	**0.010**
Trunk-to-appendicular fat ratio	0.78 [0.67, 0.83]	0.79 [0.68, 0.88]	0.739
Muscle-to-fat ratio (MFR), *z-score*	−0.47 [−0.88, −0.06]	−0.19 [−0.70, 0.21]	0.065
Blood pressure			
Systolic, *mmHg*	103 [96, 109]	103 [99, 109]	0.402
Systolic, *%*	71 [43,85]	74 [58,90]	0.122
Diastolic, *mmHg*	61 [57,67]	62 [58,66]	0.713
Diastolic, *%*	64 [48,81]	65 [46,80]	0.889
Blood pressure categories, *n (%)*			
Elevated systolic, ≥95^th^ centile	12 (20.3)	18 (28.1)	0.315
Elevated diastolic, ≥95^th^ centile	5 (8.5)	3 (4.6)	0.382
Elevated systolic and/or diastolic, ≥95^th^ centile	14 (23.7)	20 (30.8)	0.380
Glucose, *mg/dL*	84 [80,88]	86 [83, 90.8]	0.147
Lipid profile	*n* = 45	*n* = 31	
Dyslipidemia, elevated TG (TG > 110 mg/dL), *n (%)*	5 (10.6)	4 (12.5)	0.798
Dyslipidemia, low HDL (HDL < 40 mg/dL), *n (%)*	0 (0.0)	3 (9.7)	**0.033**
TG:HDL cholesterol ratio	1.07 [0.75, 1.36]	1.07 [0.90, 1.66]	0.416

Data are expressed as number (percent), mean ± standard deviation or median [interquartile range]. The delta height z-score represents the linear growth compared to the potential genetic height and it was calculated as the difference between the subjects’ height z-scores and their MPHt z-scores.

Bold values denote statistical significance at the *P* ≤ 0.05 level.

*TG* triglycerides, *HDL* high-density lipoproteins.

### Comparative analyses of children with overweight/obesity (PA vs. control, Supplementary Table 3)

The comparison between children with overweight/obesity in the PA group and those in the control group revealed similar anthropometric and body composition parameters. Children with PA and overweight/obesity exhibited higher systolic and diastolic blood pressure levels and percentiles and greater rates of elevated blood pressure (57.1% vs. 21.1%, *P* = 0.014) compared to the control group, as well as higher TG:HDL ratios (*P* = 0.026).

### Associations between body composition and MetS components (Table 5)

#### In the PA group

FATP was positively correlated with the BMI z-score (r = 0.858, *P* < 0.001), systolic BP percentile (r = 0.486, *P* < 0.001), diastolic BP percentile (r = 0.244, *P* = 0.023), TG z-score (r = 0.358, *P* = 0.002), and TG:HDL ratio (r = 0.545, *P* < 0.001). The trunk-to-appendicular fat ratio was positively correlated with the BMI z-score (r = 0.245, *P* = 0.022), TG z-score (r = 0.302, *P* = 0.010) and TG:HDL ratio (r = 0.325, *P* = 0.006). The MFR z-score was negatively correlated with the BMI z-score (r = −0.816, *P* < 0.001), systolic BP percentile (r = −0.446, *P* < 0.001), diastolic BP percentile (r = −0.226, *P* = 0.035), TG z-score (r = −0.300, *P* = 0.010), and TG:HDL ratio (r = −0.476, *P* < 0.001).

**Table 5:** Associations between body composition and metabolic syndrome components of the premature adrenarche group and their healthy controls.

	Premature adrenarche		Healthy controls	
	r	*P* value	r	*P* value
	**Fat Percentage**			
BMI, *z-score*	0.858	**<0.001**	0.774	**<0.001**
Systolic BP, *percentile*	0.486	**<0.001**	0.116	0.293
Diastolic BP, *percentile*	0.244	**0.023**	0.090	0.417
TG, *z-score*	0.358	**0.002**	−0.102	0.488
HDL, *z-score*	−0.041	0.735	0.135	0.378
TG:HDL ratio	0.325	**0.006**	0.181	0.233
	**Muscle-to-Fat ratio z-score**			
BMI, *z-score*	−0.816	**<0.001**	−0.576	**<0.001**
Systolic BP, *percentile*	−0.446	**<0.001**	−0.105	0.340
Diastolic BP, *percentile*	−0.226	**0.035**	−0.099	0.370
TG, *z-score*	−0.300	**0.010**	0.109	0.457
HDL, *z-score*	0.071	0.557	−0.220	0.146
TG:HDL ratio	−0.476	**<0.001**	0.024	0.874

Spearman correlation test applied to assess the relationship between body composition parameters and metabolic syndrome components in the PA group and in the healthy control group.

Bold values denote statistical significance at the *P* ≤ 0.05 level.

*BMI* Body mass index, *BP* blood pressure, *TG* triglyceride, *HDL* high-density lipoprotein.

#### In the healthy control group

FATP was positively correlated with the BMI z-score (r = 0.774, *P* < 0.001), and the trunk-to-appendicular fat ratio was positively correlated with the HDL z-score (r = 0.421, *P* = 0.004), while the MFR z-score was negatively correlated with the BMI z-score (r = −0.576, *P* < 0.001).

There were significant correlations between the parents’ and offsprings’ MFR findings in the control group (father-child: r = 0.528, *P* = 0.020; mother-child: r = 0.359, *P* = 0.031), no significant correlations were found in the PA group (father-child: r = 0.266, *P* = 0.0243; mother-child: r = 0.280, *P* = 0.080).

## Discussion

We conducted this observational study on prepubertal children with PA in order to determine the relationship, if any, between body composition and unfavorable metabolic outcomes. Our findings revealed, for what we believe is the first time, that prepubertal children with PA have a lower muscle-to-fat ratio compared to healthy sex- and age-matched controls. Furthermore, in subgroup analyses of PA by weight status, children with PA categorized as having overweight/obesity revealed elevated rates of high blood pressure and dyslipidemic atherogenic indices in comparison to their peers with overweight/obesity. Although we did not identify higher rates of MetS components, children with PA displayed an increased likelihood for having a family history of cardiometabolic disease. Moreover, their fat percentage and MFR z‐scores were linked with several cardiometabolic risk factors, including BP percentiles, TG concentrations, and the atherogenic dyslipidemia index (TG:HDL ratio). Taken together, the combination of their young age, family history, and unfavorable body composition could potentially place prepubertal children with PA at an increased risk of developing cardiometabolic diseases in the future.

We demonstrated that the body composition of the PA study group was characterized by higher fat indices, and that the fat percentage was positively associated with DHEAS concentrations. These findings are consistent with earlier observations of higher serum DHEAS concentrations in overweight children compared to children with normal body weight.^31,32^ Indeed, the elevated concentrations of DHEAS are considered a biochemical hallmark of PA.^4^ Body adiposity appears to affect the secretion of adrenal androgens, possibly through insulin and insulin growth factor 1, the peripheral conversion of androgen precursors to active androgens, and/or the bioavailability of androgens. This plausible mechanism is supported in the current study by the notable positive association we observed between DHEAS concentrations and HOMA-IR, a marker of insulin resistance, in the PA group. This association had been recently documented by Santos-Silva et al., and could suggest that insulin plays a role in mediating the impact of obesity on androgen production.^32^ The onset of PA may signify an early warning of metabolic disturbances induced by an unfavorable body composition leading to insulin resistance.

Our findings are in line with a study from Turkey on body composition as measured by the BIA of prepubertal girls with PA (*n* = 47), which reported fat mass higher than that of controls.^15^ Another study whose findings are in agreement with ours included 20 prepubertal girls with PA and 15 matched controls that assessed total body fat mass and fat mass in abdominal and truncal regions by means of dual-energy X-ray absorptiometry (DXA) and reported higher total fat mass and central adiposity for the study group.^16^ Our findings, however, contradict those of a study that included 15 prepubertal children with PA (12 girls) and 8 healthy controls (1 girl), which reported that the PA group had greater lean mass without differences in measures of adiposity.^17^

The etiology of premature adrenarche remains unknown.^1,33^ Several possible contributing factors have been speculated, including intra-adipose tissue activation of androgens leading to androgen excess, heritably elevated DHEAS levels, and polymorphic variations of the androgen receptor.^1,34–36^ The lack of a clear pathophysiology suggests that different etiologies may coexist, leading to subgroups within children diagnosed with PA. We speculated that children with overweight/obesity and children with a healthy weight status could represent such subgroups. Our subgroup analyses revealed higher adiposity in children with PA and a healthy weight status, with no differences in metabolic syndrome components. However, the rates of elevated blood pressure and atherogenic dyslipidemic index of the children in the overweight/obese weight category were higher in the PA group compared to controls.

We did not identify any group differences in the prevalence of elevated BP, in agreement with the findings of other reports.^17,37,38^ A recent review on premature adrenarche highlighted two important issues when addressing the impacts of PA on metabolic health. Firstly, data on cardiovascular outcomes later in life is inconclusive. Secondly, PA appears to have subtypes concerning weight and insulin sensitivity.^33^ Notably, there were strong associations between body composition parameters and both BP and the atherogenic dyslipidemia index in our PA group but not in their healthy controls. These findings underscore the importance of maintaining a favorable body composition in children with PA. Furthermore, they may suggest that although components of MetS were not found to be elevated in prepubertal children with PA, they could potentially become evident as those individuals progress through puberty and age. This holds even greater significance when considering that the children in the PA group had an increased likelihood of having a family history of cardiometabolic diseases. We propose that considering body composition could offer a potential approach to better understand the diversity within the population experiencing PA and its metabolic implications.

It is well documented that the perinatal course, including prematurity and abnormal birth weight categories, affects the risk of developing MetS later in life.^39–41^ The presence of a U-shaped relationship between birth weight and the occurrence of MetS components in childhood has also been demonstrated,^39,42^ as was the association between low birth weight with premature adrenal activation^43^ and higher concentrations of DHEAS in childhood.^32^ We did not observe any comparable group differences: most of the pregnancies were uncomplicated and had followed spontaneous conception in healthy mothers, leading to appropriate birth weights and suggesting that the in utero environment was non-contributory. Importantly, we also did not observe any associations between perinatal characteristics and MetS components. Therefore, it would be reasonable to assume that the weight gain during early childhood and its impact on body composition in the children of our PA group were more likely influenced by exposure to postnatal factors.

### Limitations and strengths

We acknowledge limitations to this study that bear mention. It is mainly limited by its retrospective design, which does not allow us to establish causality between the manifestation of PA and the unfavorable body composition that we observed in the children who comprised our PA group. Another limitation is the lack of HOMA-IR and HbA1c data, as well as DHEAS levels for the control group, although PA data were compared to sex- and age-matched values in European children. Furthermore, the self-reporting of family history may have introduced bias. The strength of our study lies in the relatively large cohort of patients with PA and the availability of BIA samples for all of the participants. specifically, the number of boys in a field that is mainly researched on girls. Another strength lies in the uniformity of medical care provided by the same multi-professional team in a single hospital-based tertiary center and the comprehensiveness of patient follow-up. Finally, the use of healthy controls and the calculation of sex- and age-adjusted z-scores/percentiles allows for comparisons between subjects and more robust interpretation of the data.

## Conclusions

While the children in our PA group did not exhibit higher rates of MetS components compared to their healthy controls, it is possible that such abnormalities may manifest at a later stage in their lives, with their unfavorable body composition acting as a potential risk factor in their development. Heritable body composition traits, such as the muscle-to-fat ratio, were demonstrated only in healthy children but not in those with PA, suggesting environmental influence on their development. Our findings raise the possibility that body composition may have an influence on metabolic syndrome components in prepubertal children with PA. These findings underscore the importance of monitoring these children and alert for the potential need for early intervention strategies to improve body composition with the aim of mitigating the risk of developing cardiometabolic diseases. Further research is warranted to elucidate the trajectory of body composition acquisition in the context of PA and its implications for metabolic health.

## Supplementary information

Supplementary Matrerial

## Data Availability

The data used to support the findings of this study are available from the corresponding author upon reasonable request.
