# Supplementary material for: Body composition in prepubertal children with idiopathic premature adrenarche: implications for cardiometabolic health
Source: Pediatr Res. 2024 Dec 18;98(2):663–71. doi: 10.1038/s41390-024-03776-2 (PMC12454123; doi:10.1038/s41390-024-03776-2)
Supplement: Supplementary file 1 — Supplementary Matrerial [file 41390_2024_3776_MOESM1_ESM.pdf]

Supplementary Table 1
Pregnancy And Perinatal Characteristics of The Premature Adrenarche Group And Their Healthy Controls

	**Premature Adrenarche**	**Healthy Controls**	***P* value**
Conception, *n (%)*	n=87	n=85	
Spontaneous	64 (73.5)	72 (84.7)	.315
Ovulation induction	1 (1.2)	3 (3.5)	
Intrauterine insemination	8 (9.2)	2 (2.4)	
IVF	7 (8.0)	4 (4.7)	
IVF sperm donation	3 (3.4)	3 (3.5)	
IVF egg donation	2 (2.3)	1 (1.2)	
Surrogate	1 (1.2)	0 (0.0)	
Adoption	1 (1.2)	0 (0.0)	
Maternal conditions, *n (%)*	n=85	n=85	
Gestational diabetes mellitus	7 (8.2)	6 (7.1)	.787
Polycystic ovarian syndrome	8 out of 83 (9.6)	7 out of 85 (8.2)	.751
Fetus, *n (%)*	n=87	n=87	
Twin	8 (9.2)	5 (5.7)	.387
Mode of delivery, *n (%)*	n=85	n=85	
Spontaneous vaginal	60 (70.6)	61 (71.8)	.479
Vacuum extraction	2 (2.4)	3 (3.5)	
Elective cesarean section	16 (18.8)	10 (11.8)	
Urgent cesarean section	7 (8.2)	11 (12.9	
GA	n=86	n=83	
GA, weeks [IQR]	39 [38, 40]	39 [38, 40.1]	.512
Preterm, <37 wks	13 (15.1)	11 (12.9)	.823
Term, 38-42 wks	70 (81.4)	72 (84.7)	
Postterm, ≥42 wks	3 (3.5)	2 (2.4)	
Birth parameters	n=83	n=85	
Birth weight, *grams*	3000 [2700, 3350]	3110 [2700, 3480]	.458
Birth weight, *z-score*	-0.410 [-0.960, 0.245]	-0.330 [-0.877, 0.292]	.713
SGA, *n (%)*	13 (15.7)	10 (11.9)	.701
AGA, *n (%)*	66 (79.5)	71 (84.5)	
LGA, *n (%)*	4 (4.8)	3 (3.6)	

Data are expressed as number and (percent) of valid cases or median [interquartile range].
Bold values denote statistical significance at the *P* ≤.05 level.
Abbreviations: GA, gestational age; SGA, small for gestational age; AGA, appropriate for gestational age; LGA, large for gestational age; IFV, in vitro fertilization; IQR, interquartile range.

Supplementary Table 2
Laboratory Evaluation

	Premature adrenarche	Healthy controls	*P* value
Glucose, *mg/dL*	85 [80, 89]	86 [82, 90]	.413
Hemoglobin A1c, *%*	5.4 ± 0.3		
Insulin, *mU/L*	10.9 ± 6.2		
HOMA-IR	2.4 ± 1.3		
Cholesterol, *mg/dL*	168 ± 27	164 ± 27	.446
Cholesterol, z-scores	0.26 ± 0.95	0.13 ± 1.0	.461
Triglycerides, *mg/dL*	71 [52, 96]	65 [51, 80]	.381
Triglycerides, *z-scores*	-0.07 ± 1.04	-0.22 ± 0.91	.432
High-density lipoprotein cholesterol, *mg/dL*	56 ± 11	55 ± 14	.550
High-density lipoprotein cholesterol, *z-scores*	0.40 ± 0.89	0.25 ± 1.19	.441
Low-density lipoprotein cholesterol, *mg/dL*	96 ± 23	96 ± 22	.984
Low-density lipoprotein cholesterol, z-scores	0.11 ± 1.03	0.13 ± 0.96	.915
Non-high-density lipoprotein cholesterol, *mg/dL*	112 ± 25	107 ± 24	.331
Non-high-density lipoprotein cholesterol, *z-scores*	0.16 ± 1.03	-0.03 ± 0.99	.332
Thyroid-stimulating hormone, *mIU/L*	2.31 [1.55, 3.01]	2.06 [1.47, 2.67]	.184
Free T4, *pmol/L*	15.56 ± 1.97	15.66 ± 1.56	.814

Data are expressed as number and (percent) or median [interquartile range]. **Bold** values denote statistical significance at the *P* ≤.05 level.
Abbreviations: HOMA-IR, Homeostatic Model Assessment for Insulin Resistance

Supplementary Table 3
Subgroup Analyses within Overweight/obese Weight Status between the Premature Adrenarche Group and Healthy Control Group

	Overweight/obese PA *n*=28	Overweight/obese HC *n*=19	*P* value
Girls (%)	21 (75)	16 (84.2)	.449
Age, years	8.2 ± 1.4	8.0 ± 1.2	.562
Anthropometrics			
Height, *z-score*	1.05 [0.43, 1.50]	0.78 [0.08, 1.45]	.398
Mid-parental height (MPHt), *z-score*	0.11 ± 0.86	0.29 ± 0.86	.495
Delta height, *z-score*	0.92 ± 0.97	0.55 ± 0.82	.198
Body mass index, *z-score*	1.73 [1.54, 1.91]	1.52 [1.39, 1.96]	.595
Body composition parameters			
Fat, *percentage*	31.3 [21.2, 35.1]	31.5 [29.6, 33.9]	.914
Truncal fat mass, *%*	25.6 [21.5, 29.3]	25.8 [23.9, 29.0]	.688
Truncal fat mass, *kg*	5.8 [4.0, 6.8]	5.3 [4.6, 6.4]	.837
Appendicular fat mass, *kg*	6.7 [5.1, 8.4]	5.8 [5.0, 7.8]	.340
Trunk-to-appendicular fat ratio	0.83 [0.74, 0.86]	0.86 [0.84, 0.92]	**.020**
Muscle-to-fat ratio (MFR), *z-score*	-1.36 [-1.59, -1.18]	-1.52 [-1.79, -1.14]	.404
Blood pressure			
Systolic, *mmHg*	113 [109, 116]	105 [98, 112]	**.003**
Systolic, *%*	91 [83, 94]	75 [60, 86]	**.023**
Diastolic, *mmHg*	69 [65, 71]	61 [57, 66]	**.009**
Diastolic, *%*	83 [64, 86]	57 [45, 76]	**.027**
Blood pressure categories, *n (%)*			
Elevated systolic, ≥95th centile	16 (57.1)	4 (21.1)	**.014**
Elevated diastolic, ≥95th centile	6 (21.4)	3 (15.8)	.630
Elevated systolic and/or diastolic, ≥95th centile	16 (57.1)	6 (31.6)	.085
Glucose, *mg/dL*	88 [82, 90]	87 [80, 89]	.483
Lipid profile	n=25	n=15	
Dyslipidemia, elevated TG (TG >110 mg/dL), *n (%)*	7 (28)	1 (6.7)	.102
Dyslipidemia, low HDL (HDL<40 mg/dL), *n (%)*	4.0 (16.0)	1 (14.3)	.887
TG:HDL cholesterol ratio	1.72 [1.49, 2.37]	1.29 [1.13, 1.47]	**.026**

Data are expressed as number (percent), mean ± standard deviation or median [interquartile range]. The delta height z-score represents the linear growth compared to the potential genetic height and it was calculated as the difference between the subjects' height z-scores and their MPHt z-scores. **Bold** values denote statistical significance at the *P* ≤.05 level. Abbreviations: TG, triglycerides; HDL, high-density lipoproteins
